# The Profession of British Nurses: II. Plain Words on Registration


**Published:** 1917-04-07

**Authors:** 


### THE MATRONS' AND SISTERS' DEPARTMENT.

### THE PROFESSION OF BRITISH NURSES.

### II. Plain Words on Registration.*

THE MEDICAL ACT.

In endeavouring to measure with approximate accuracy the benefits likely to accrue to nurses from State Registration it is desirable to examine what the State has actually done in this connection concerning other professions. British legislation is wisely conservative and proceeds "from precedent to precedent." Hence it is safe to assume that what has already been done by Parliament in establishing State Registers will, speaking generally, be followed in the case of nurses.

Closely allied to the nursing profession, and in constant association with it, is that of medicine. The famous Act of 1858, then known as the Medical Act, established the General Medical Council, and set up machinery for examining and registering medical practitioners. This was not the first State effort to register properly qualified physicians, for in the days of the Stuarts Royal Charters were granted to certain colleges and training-schools. But it was the greatest and the most effectual. It will be found on examination of the provisions of this Act of Parliament that the sole object of the Legislature in giving effect to it was the protection of the public, and not of the medical profession. This is by no means generally understood, and it is of vital importance, especially for nurses, to appreciate that it is no part of the functions of the Legislature to protect any profession or trade, no matter how deserving. It may so happen, but it does not necessarily follow, that in protecting the public the members of a profession may also receive protection. The preamble to the Medical Act opens thus: "Whereas it is expedient that persons requiring medical aid should be enabled to distinguish qualified from unqualified practitioners." This at once defines the motive of the Legislature. As we proceed further the negative attitude of the State towards the profession becomes more evident. The Act imposes no penalty nor sets any restriction on the practice of empirics or quacks, and as a matter of fact there is nothing in English law to prevent any person whomsoever from practising medicine and receiving fees. It will be found, indeed, and this we hope to demonstrate, that in no case does the State take to itself the function of establishing anything corresponding with a trade union. The public are given an opportunity of identifying without trouble the hall-marked article, and after this is done the liberty of the subject is fully recognised.

A like principle governs appointments. Public appointments—that is to say, appointments paid for out of public moneys—are strictly limited by this Act to persons whose names appear on the Register, but there is nothing in the law to prevent private persons from giving salaried appointments to unqualified medical practitioners. "No person," runs the text, "shall hold any appointment as a physician, surgeon, or other medical officer, either in the Military or Naval Service, or in emigrant or other vessels, or in any hospital, infirmary, dispensary, or lying-in hospital not supported wholly by voluntary contributions, or in any lunatic asylum, gaol, penitentiary, house of correction, house of industry, parochial or union workhouse, or poor-house, parish union, or other public establishment." This list is carefully selected, and the words of the Act make very clear that the protection of the public, and this only, concerned the Legislature. Similarly there are clauses in this Act which render it impossible for any but registered persons to recover fees, or for any but registered persons to issue medical certificates "required by any Act now in force or that may hereafter be passed."

In the 'fifties, when the Medical Act was passed, education was in a very backward condition, and the masses who now read their morning papers were illiterate. They were preyed upon by a horde of unscrupulous and incompetent vampires, who took advantage of their ignorance and their illness. If the circumstances which now prevail then existed there would not have been any pressing necessity for legislation, and nurses may regard it as true that the main cause of the delay in passing a Registration Act for them is, that there is little, if any, public necessity for it. The regulations governing the appointment of nurses in the Navy, the Army, in public institutions of all kinds, including Poor-Law infirmaries and municipalities, prescribe that fully-trained nurses only shall be eligible.

A useful exercise for the intelligent nurse, in view of all the storms that rage round the subject of Registration, would be to assume that an Act of Parliament called the Nurses Act, whose provisions correspond with those of the Medical Act, were immediately to be put in force. If such an Act were law to-morrow, what would it profit her? Would the market be less crowded, would salaries automatically rise, would the hours of toil diminish? These are questions which might profitably engage the attention of the champions of Registration.

* For first article see The Hospital, March 31, p. 521.

